# Single-cell RNA sequencing reveals chondrocyte heterogeneity and fibrocartilage transition in osteonecrosis of the femoral head

**DOI:** 10.1016/j.isci.2026.116724

**Published:** 2026-08-22

**Authors:** Tun Liu, Kai Nan, Xiaoqian Dang, Wei Wang, Jidong Song

**Affiliations:** 1 Department of Orthopedics, The Second Affiliated Hospital, Xi’an Jiaotong University, Xi’an, Shaanxi Province, China; 2 Department of Joint Surgery, Honghui Hospital, Xi’an Jiaotong University, Xi’an, Shaanxi Province, China

**Keywords:** osteonecrosis of the femoral head, ONFH, single-cell RNA sequencing, scRNA-seq, chondrocytes, fibrocartilage, intercellular communication

## Abstract

Cartilage degeneration in osteonecrosis of the femoral head (ONFH) is closely associated with chondrocyte heterogeneity. Single-cell RNA sequencing was performed on 50,851 chondrocytes from 10 ONFH patients and 10 controls. The findings were further validated using an *in vitro* chondrocyte injury model induced by dexamethasone. Nine chondrocyte subtypes were identified, among which COL1A1⁺ chondrocytes (exhibiting fibrotic features and Hippo-YAP pathway activation) and COL2A1⁺ chondrocytes (displaying a reparative hyaline cartilage phenotype) were significantly enriched in ONFH. Immunohistochemistry and *in vitro* functional assays confirmed the upregulation of signature proteins and key transcription factors in these subtypes. Pseudotime analysis revealed that CCL20⁺ progenitor cells differentiate toward fibrotic or reparative fates via PRG4 or SOX5, respectively. Key ligand-receptor pairs, including FGF2-FGFR1 and TNFRSF11B-TNFSF11, mediated intercellular communication. This study elucidates the cellular and molecular basis of the “fibrosis-repair” imbalance in ONFH cartilage degeneration, offering a theoretical foundation for targeting chondrocyte fate regulation.

## Highlights

• ONFH cartilage contains COL1A1^+^ fibrotic and COL2A1^+^ reparative chondrocyte subpopulations
• PRG4 and SOX5 drive fibrotic and reparative differentiation, respectively
• RUNX2 drives hypertrophy and matrix degradation in the COL1A1^+^ subpopulation
• FGF2-FGFR1 and OPG-RANKL mediate key cellular signaling

## Graphical abstract

**Figure:**
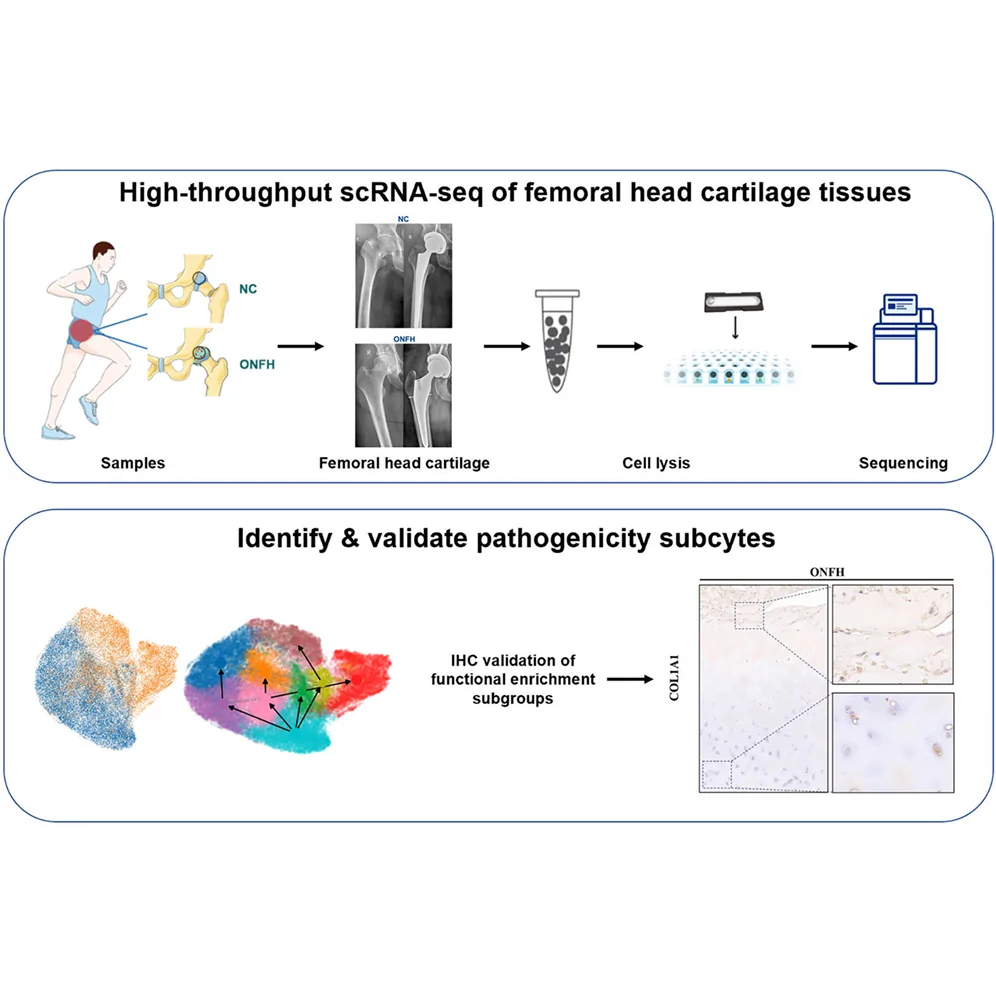

## Teaser

Biological sciences

## Introduction

Osteonecrosis of the femoral head (ONFH) is a debilitating orthopedic disorder marked by femoral head collapse and subsequent osteoarthritis, which often results in chronic pain, limited mobility, and impaired joint function.^1^^,^^2^ Articular cartilage, an avascular tissue with limited self-renewal capacity, is primarily composed of chondrocytes embedded within an extracellular matrix (ECM). Degeneration of this cartilage layer is known to accelerate ONFH progression.^3^^,^^4^ In younger patients, hip-preserving interventions may be improved by delaying cartilage degradation, which often follows bone reconstruction and the onset of secondary osteoarthritis.^5^ Thus, elucidating the pathogenic mechanisms mediated by chondrocytes in ONFH is essential for developing targeted therapies.

Articular cartilage develops from mesenchymal stem cells (MSCs) through chondrogenesis, a differentiation process accompanied by the secretion of a collagen-based ECM.^6^^,^^7^ Several subtypes of chondrocytes have been identified in articular cartilage, including proliferative chondrocytes (ProCs), prehypertrophic chondrocytes (preHTCs), and hypertrophic chondrocytes (HTCs).^8^ ProCs are predominantly located in the growth plates’ proliferative zone. PreHTCs can control the start of hypertrophic differentiation. And HTCs can manage the mineralization of the cartilage matrix. Recently, cartilage progenitor cells (CPCs) were discovered. These cells are recognized for their self-renewal capacity, differentiate into multiple lineages, express stem-cell-related markers, and contribute to the repair and homeostasis of ONFH cartilage.^8^^,^^9^^,^^10^ Traditionally, chondrocyte subtypes were categorized based on their developmental origin, location, physical properties, morphology, and molecular functions. However, due to the phenotypic variability and the scarcity of markers for identification, isolation, and manipulation, the classification of articular cartilage chondrocytes in human ONFH remains undetermined. These uncertainties impact our understanding of the development and function of each chondrocyte group in ONFH pathogenesis.

Global gene expression profiling has identified mechanisms underlying ONFH. However, bulk omics analyses, in which average expression change across heterogeneous tissues, may obscure ONFH-related alterations specific to certain cell subtypes. Consequently, the composition of ONFH cartilage cell types and the cellular heterogeneity driving disease progression remain largely unexplored.^3^^,^^11^

Single-cell RNA sequencing (scRNA-seq) offers high-resolution transcriptomic profiling at the cellular level, providing an opportunity to investigate chondrocyte heterogeneity in ONFH cartilage.^12^ This study aimed to characterize molecular programs governing cell population relationships by performing scRNA-seq analyses of healthy and ONFH human hip cartilage.

## Results

### Single-cell transcriptomic landscape of femoral cartilage identifies ONFH-specific chondrocyte subsets and their functional signatures

**Figure 1:**
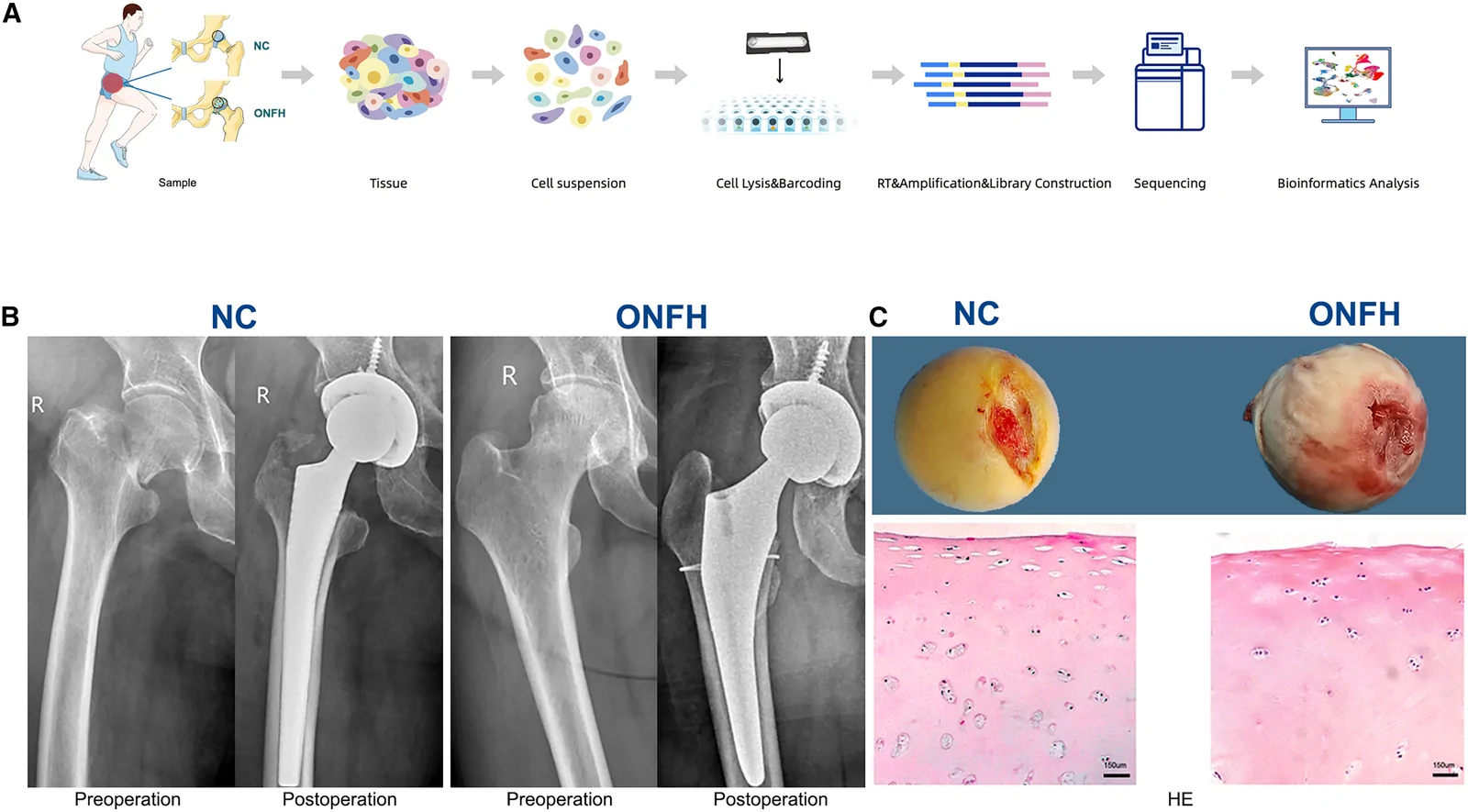
Schematic workflow and basic characterization of femoral head samples (A) Schematic workflow followed in this study. (B) The X-ray of the NC and ONFH hip joint before and after THA. (C) The imaging of the femoral head from NC and ONFH patients and the hematoxylin and eosin (HE) of the NC and ONFH cartilage. Scale bars, 150 μm.

**Figure 2:**
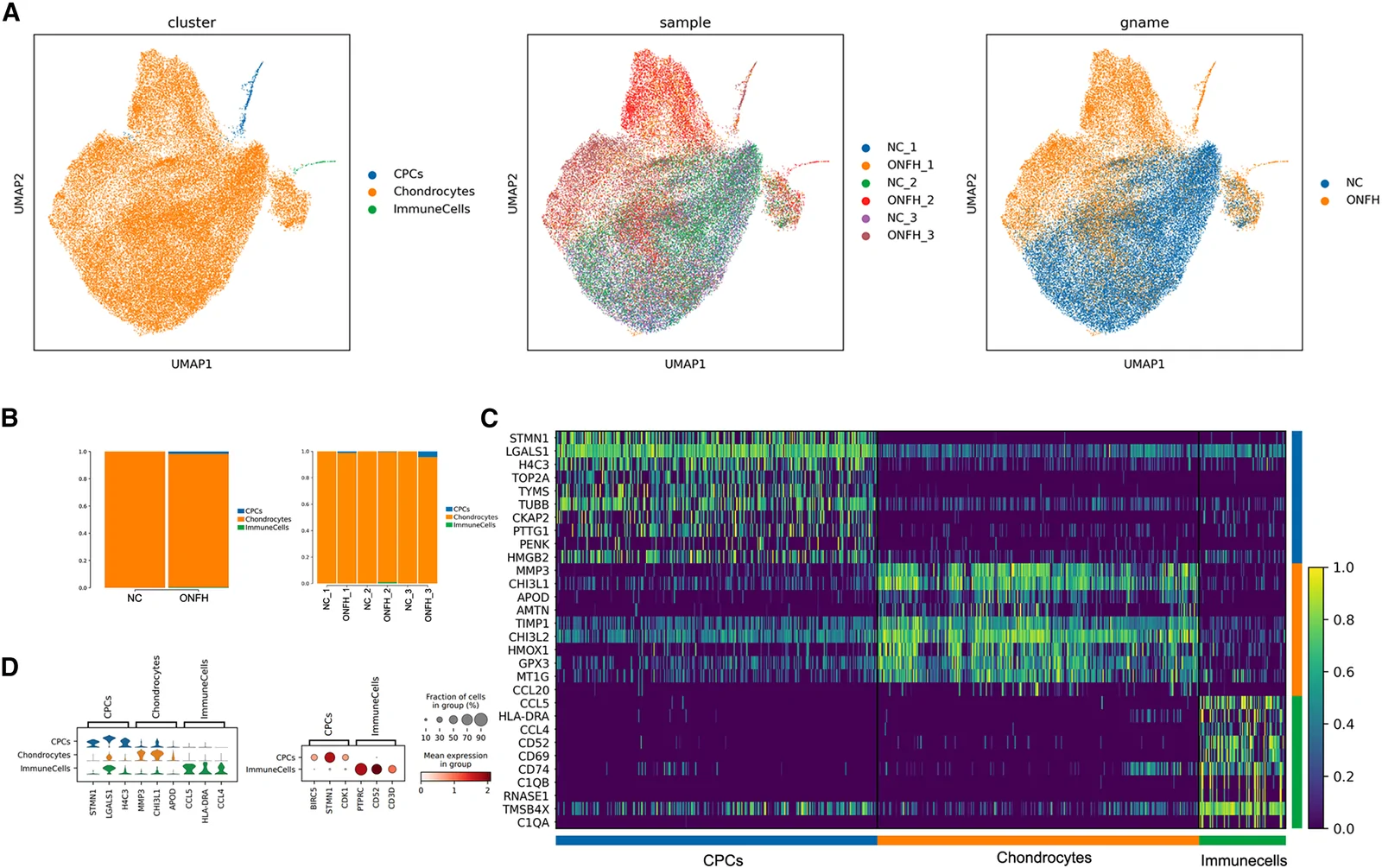
Characterization of cartilage cell changes in femoral heads (A) Uniform manifold approximation and projection (UMAP) plot showing 50,851 single cells from normal control and ONFH femoral head cartilage, colored by cell type. Three major cell lineages were identified: chondrocytes (green), cartilage progenitor cells (CPCs, orange), and immune cells (purple). (B) Bar plots showing the proportion of each cell type across individual samples (left) and aggregated by group (right). Chondrocytes constituted the predominant population in both NC and ONFH groups. (C) Heatmap displaying the top 10 differentially expressed genes (DEGs) for each cell type, ranked by log_2_ (fold change). (D) The top3 most significantly different genes (left) and top3 marker genes dot plot (right) across cell types. Dot plots showing the top3 marker genes for each cell type. Dot size represents the percentage of cells expressing the gene; color intensity indicates average expression level.

Using single-cell analysis, we constructed cell atlases comprising 50,851 cells across three cell types: chondrocytes, CPCs, and immune cells from the specified groups (Figures 1 and 2A). Chondrocytes predominated the cellular composition in each group (Figure 2B), suggesting the absence of confounders in sample collection and processing. Differential gene analysis and correlation analysis were conducted for each cell type. The heatmap in Figure 2C presented the top 10 differential genes, organized by log-fold changes, with the top3 marker genes highlighted in Figure 2D. Notably, genes such as *Stmn1*, *Ptprc*, and *CD52* exhibited close associations with CPCs and immune cells, underscoring their potential involvement in the pathogenesis of ONFH.

**Figure 3:**
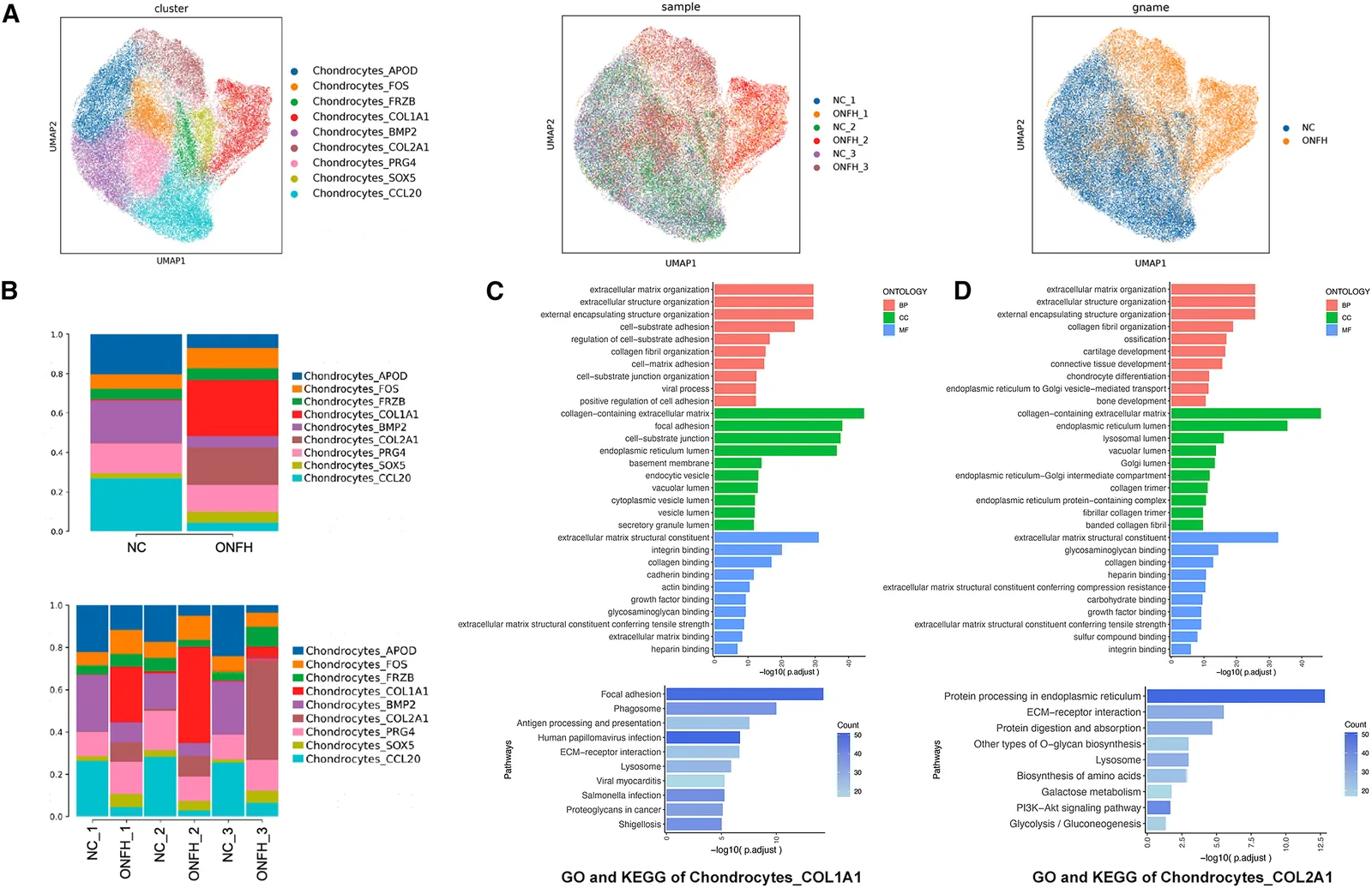
Single-cell transcriptional profiling and related subpopulation analysis of chondrocytes (A) UMAP plot of 50,388 chondrocytes re-clustered into nine distinct subpopulations, colored and labeled by their top marker genes. (B) Stacked bar plots showing the proportion of each chondrocyte subpopulation across individual samples (up) and aggregated by group (down). (C) GO enrichment analysis (top 30 terms) for Chondrocytes_COL1A1 and KEGG enrichment analysis (top 10 terms) for Chondrocytes_COL1A1. (D) GO enrichment analysis (top 30 terms) for Chondrocytes_COL2A1 and KEGG enrichment analysis (top 10 terms) for Chondrocytes_COL2A1.

Next, various subgroups of chondrocytes were examined. A total of 50,388 chondrocytes were obtained through scRNA-seq and subdivided into nine subtypes: Chondrocytes_APOD, Chondrocytes_FOS, Chondrocytes_FRZB, Chondrocytes_COL1A1, Chondrocytes_BMP2, Chondrocytes_COL2A1, Chondrocytes_PRG4, Chondrocytes_SOX5, and Chondrocytes_CCL20. Analysis of the proportions of each sample within these subgroups (Figures 3A and 3B) revealed a higher percentage of Chondrocytes_COL1A1 in the ONFH_1 and ONFH_2 groups, and a higher percentage of Chondrocytes_COL2A1 in the ONFH_3 samples compared to each NC group. Overall, the proportions of Chondrocytes_APOD and Chondrocytes_CCL20 were significantly lower in the ONFH group, whereas the proportions of Chondrocytes_COL1A1 and Chondrocytes_COL2A1 were significantly higher. The cellular occupancy ratio between the sample groups showed that the cell types relatively specific to the ONFH group were Chondrocytes_COL1A1 and Chondrocytes_COL2A1. This finding was consistent with other studies: Chondrocytes_COL1A1 specifically upregulate fibroblast-associated genes such as *COL1A1*, *COL1A2*, *MMP2*, *S100A4*, *COL3A1*, and *POSTN*, which are associated with fibrochondrocytes. Chondrocytes_COL2A1 specifically upregulate genes such as *COL2A1*, *CILP*, *CHAD*, *COL9A3*, *COMP*, and *FMOD*, which are associated with reparative cartilage.^6^

We further conducted the functional enrichment analysis of these two unique subgroups. The top 30 significantly enriched pathways were selected and represented using bar graphs (Figures 3C and 3D; Figure S3). The results showed that the Chondrocytes_COL1A1 subgroup exhibited significant enrichment in Kyoto Encyclopedia of Genes and Genomes (KEGG) pathways that upregulated focal adhesion, phagosome, and antigen processing and presentation pathways. The Chondrocytes_COL2A1 subgroup showed enrichment in KEGG pathways related to protein processing in the endoplasmic reticulum, ECM-receptor interaction, and protein digestion and absorption. Additionally, both subgroups exhibited GO enrichment associated with ECM regulation. The ECM of articular cartilage is crucial for maintaining the chondrocytes’ survival environment, providing support and nutrition, and facilitating signal exchange between chondrocytes and their surroundings. Based on these findings, we hypothesized that the loss of the chondrocyte ECM during ONFH could lead to the mechanical degeneration of articular cartilage, deformation of the joint structure, and ultimately induce ONFH.^13^

### Spatial and *in vitro* validation of COL1A1 fibrotic and COL2A1 reparative chondrocytes

**Figure 4:**
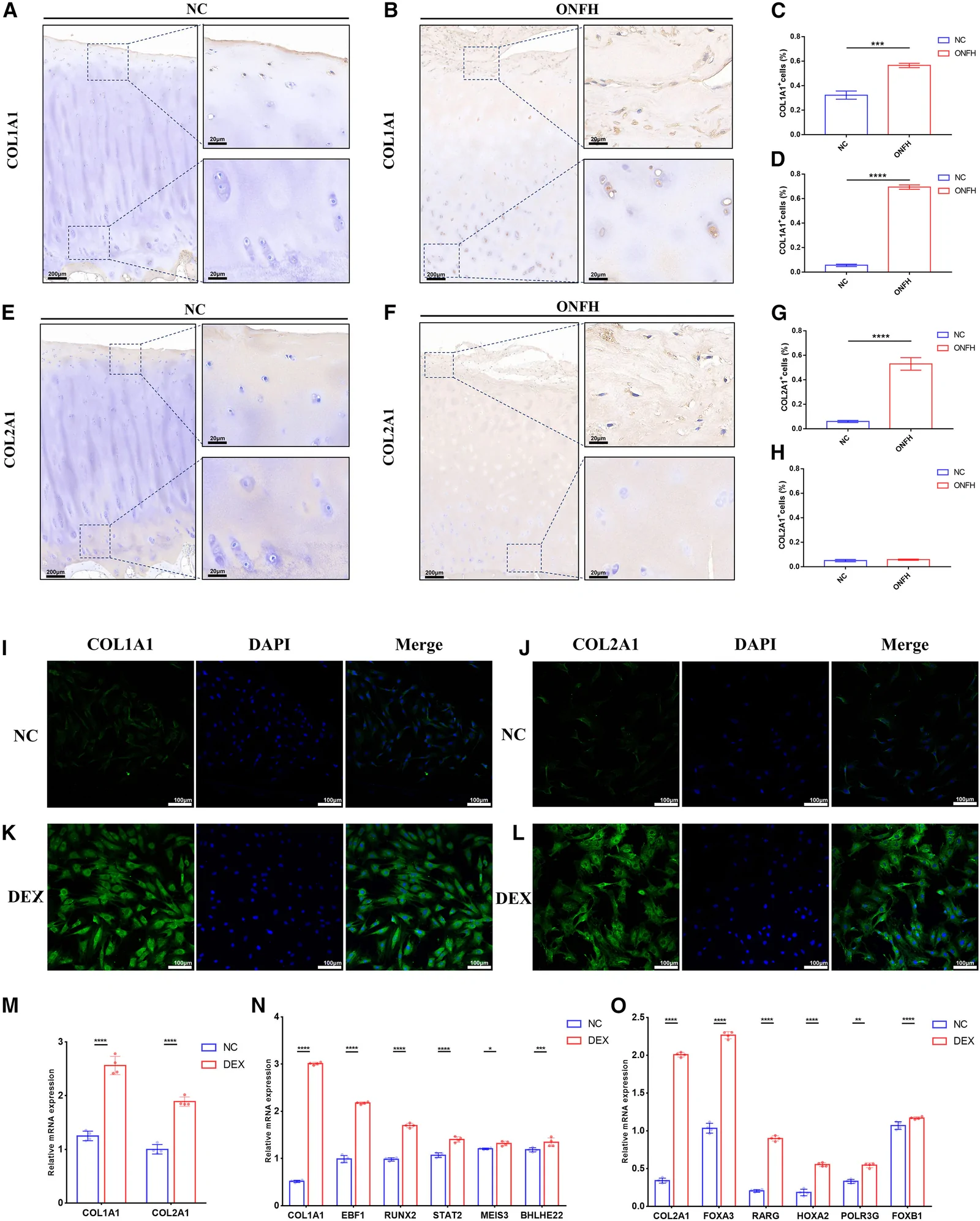
Representative immunohistochemical determinations of the indicated genes in cartilage tissues (A–D) Representative immunohistochemical determinations of COL1A1 (A and B) in cartilage tissues of the femoral head of normal human and ONFH patients. Fractions of superficial (C) and deep (D) COL1A1 genes in cartilage tissues based on immunohistochemical determinations are shown. Data shown are mean ± SEM (*n* = 3). ns, no significant difference; ∗∗∗*p* < 0.001 and ∗∗∗∗*p* < 0.0001. Scale bars, 200 μm (left) and 20 μm (right). (E–H) Representative immunohistochemical determinations of COL2A1 (E and F) in femoral head cartilage tissues from normal human and ONFH patients. Fractions of upper (G) and lower (H) COL2A1 genes in cartilage tissues based on immunohistochemical assays are shown. Scale bars, left, 200 μm; right, 50 μm; ∗∗∗*p* < 0. 001 and ∗∗∗∗*p* < 0. 0001, otherwise, not significant. Data shown are mean ± SEM (*n* = 3). ns, no significant difference; ∗∗∗∗*p* < 0.0001. Scale bars, 200 μm (left) and 20 μm (right). (I–O) *In vitro* validation using a dexamethasone (DEX)-induced rat chondrocyte injury model. Primary rat chondrocytes were treated with vehicle (control) or 10^−4^ M DEX for 48 h. Representative immunofluorescence images of COL1A1 (I and K) and COL2A1 (J and L) in control and DEX-treated chondrocytes. Scale bars, 100 μm. Quantification of mean fluorescence intensity for COL1A1 and COL2A1(M). Quantitative real-time PCR analysis of transcription factors enriched in the Chondrocytes_COL1A1 subpopulation (N) and in the Chondrocytes_COL2A1 subpopulation (O). Data shown is mean ± SEM (*n* = 3). ns, no significant difference; ∗*p* < 0.05, ∗∗*p* < 0.01, ∗∗∗*p* < 0.001, and ∗∗∗∗*p* < 0.0001. Scale bars, 100 μm.

To validate our single-cell sequencing findings, we conducted systematic immunohistochemical analyses of COL1A1 and COL2A1 expression patterns across distinct cartilage zones in normal human femoral heads and ONFH specimens (Figure 4). Quantitative evaluation revealed striking spatial and pathological variations in collagen expression profiles. In deep cartilage layers, COL1A1 exhibited minimal expression in normal controls but showed significant elevation in ONFH specimens (*p* < 0.0001; Figures 4A, 4B, and 4D). While superficial cartilage demonstrated baseline COL1A1 expression in both groups, ONFH samples displayed marked upregulation compared to normal tissues (*p* < 0.001; Figures 4A–4C). Conversely, COL2A1 expression patterns displayed zonal specificity, with superficial chondrocytes in ONFH specimens showing significant overexpression (*p* < 0.0001; Figures 4E–4G), while deep cartilage layers maintained comparable low expression levels between groups (Figures 4E, 4F, and 4H).

To further validate these findings and corroborate the transcriptional programs identified by scRNA-seq, we established an *in vitro* ONFH chondrocyte injury model using primary rat chondrocytes treated with 10^−4^ M dexamethasone (DEX) for 48 h (Figures 4I–4O). Immunofluorescence staining, combined with quantitative analysis of mean fluorescence intensity, revealed that DEX treatment significantly upregulated the protein expression of Chondrocytes_COL1A1 and Chondrocytes_COL2A1 (Figures 4I–4M; ∗∗∗∗*p* < 0.0001). These results are consistent with the aberrant expression patterns observed in clinical specimens (Figures 4A–4H) and our scRNA-seq data (Figure 3), thereby reinforcing the pathological relevance of the identified chondrocyte subpopulations. To assess the functional activity of the core transcriptional regulatory networks inferred from regulon specificity score (RSS) analysis under pathological conditions, we examined the expression of subpopulation-enriched transcription factors in the DEX-induced injury model. Quantitative real-time PCR analysis demonstrated that DEX treatment significantly upregulated transcription factors specifically enriched in the Chondrocytes_COL1A1 subpopulation—including EBF1, Runx2, Stat2, Meis3, and Bhlhe22—consistent with the activation of a pro-fibrotic, catabolic transcriptional program (Figure 4N). Conversely, DEX treatment also led to a significant increase in the expression of transcription factors enriched in the Chondrocytes_COL2A1 subpopulation, such as Foxa3, Rarg, Hoxa2, Polr3g, and Foxb1 (Figure 4O), supporting the induction of a reparative transcriptional program.

Collectively, these findings demonstrate that the distinct transcriptional regulatory networks delineated by our scRNA-seq analysis are reproducibly activated in an independent *in vitro* model of femoral head necrosis. This provides functional validation of key molecular signatures associated with two major disease-relevant chondrocyte lineages.

### Pseudotime trajectory and transcriptional networks driving chondrocyte fate decisions in ONFH

Pseudotemporal analysis, a well-established tool in scRNA-seq analysis, elucidates sequential changes in gene expression during cell state transitions. In this study, pseudotemporal analysis was employed to explore the differentiation patterns of chondrocyte subpopulations. The analysis revealed that the Chondrocytes_APOD and Chondrocytes_CCL20 subgroups differentiate toward the Chondrocytes_COL1A1 and Chondrocytes_COL2A1 subgroups, respectively, marking the developmental starting points. Specifically, the Chondrocytes_CCL20 subgroup demonstrated the upregulation of CCL20, TNFAIP6, and MMP1, markers associated with HTCs, consistent with previous studies.^14^ The PAGA trajectory extrapolation showed that chondrocyte subtypes within the ONFH group terminated in Chondrocytes_COL1A1 and Chondrocytes_COL2A1 states via two developmental routes: Chondrocytes_CCL20-Chondrocytes_SOX5-Chondrocytes_COL1A1 and Chondrocytes_CCL20-Chondrocytes_PRG4-Chondrocytes_COL1A1. In contrast, the NC group followed three distinct developmental routes: CCL20-BMP2-APOD, CCL20-PRG4-FOS, and CCL20-FRZB.

**Figure 5:**
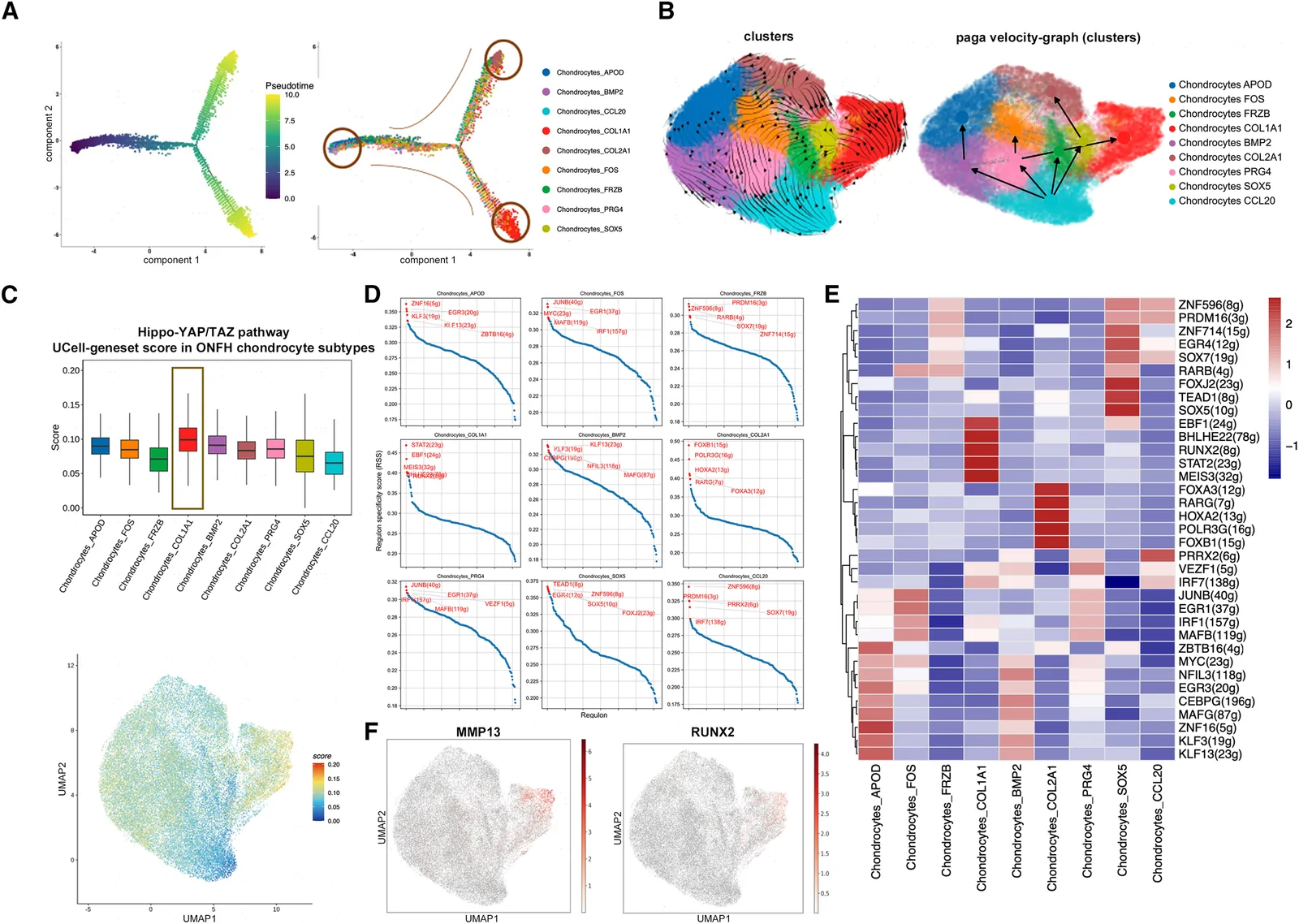
Transcriptional dynamics and regulatory networks of chondrocyte subpopulations in ONFH (A) Pseudotemporal analysis to explore differentiation patterns of chondrocytes subgroups. Cells transition from a progenitor-like state (Chondrocytes_CCL20, left) toward two distinct terminal fates: Chondrocytes_COL1A1 (fibrogenic branch) and Chondrocytes_COL2A1 (reparative branch). (B) Velocity graphs predicting developmental routes. Arrow thickness indicates transition confidence. ONFH samples displayed two dominant trajectories: CCL20→SOX5→COL1A1 and CCL20→PRG4→COL1A1. (C) UCell gene set enrichment scores for the Hippo-YAP/TAZ signaling pathway across chondrocyte subpopulations. (D) Heatmap of RSS identifying the top five transcription factors with the highest regulatory specificity for each chondrocyte subpopulation. (E) Heatmap comparing regulon activity of key transcription factors between nine chondrocyte subgroups. (F) Specific expression of RUNX2 and its target gene MMP13 in Chondrocytes_COL1A1 compared to other subpopulations.

Regulons are collections of genes regulated by the same regulatory element (transcription factor). Each regulon can be defined as a collection of transcription factors and their target genes. The RSS and regulon activity were utilized to further explore the transcriptomic characteristics of chondrocyte subtypes in ONFH. A higher RSS score indicates a more prominent regulatory role of the transcription factor in a specific cell population. The top five RSS scores for each chondrocyte subtype were highlighted in Figure 5D. Figure 5E compares regulator activity between groups, identifying MEIS2 (12 g), BHLHE22 (78 g), RUNX2 (8 g), STAT2 (23 g), and EBF1 (32 g) as having higher regulatory activity in the Chondrocytes_COL1A1 subgroup. Additionally, FOXA3 (12 g), RARG (7 g), HOXA2 (13 g), POLR3G (16 g), and FOXB1 (15 g) exhibit high activity in the Chondrocytes_COL2A1 subgroup. RUNX2, a key transcription factor involved in chondrocyte hypertrophy, was confirmed to be highly active in the Chondrocytes_COL1A1 subgroup, along with its target gene, MMP13, as shown in Figure 5F. Moreover, the Hippo-YAP/TAZ pathway was significantly upregulated in Chondrocytes_COL1A1 compared to other subtypes (Figure 5C).

### Intercellular communication and metabolic heterogeneity of chondrocyte subpopulations

**Figure 6:**
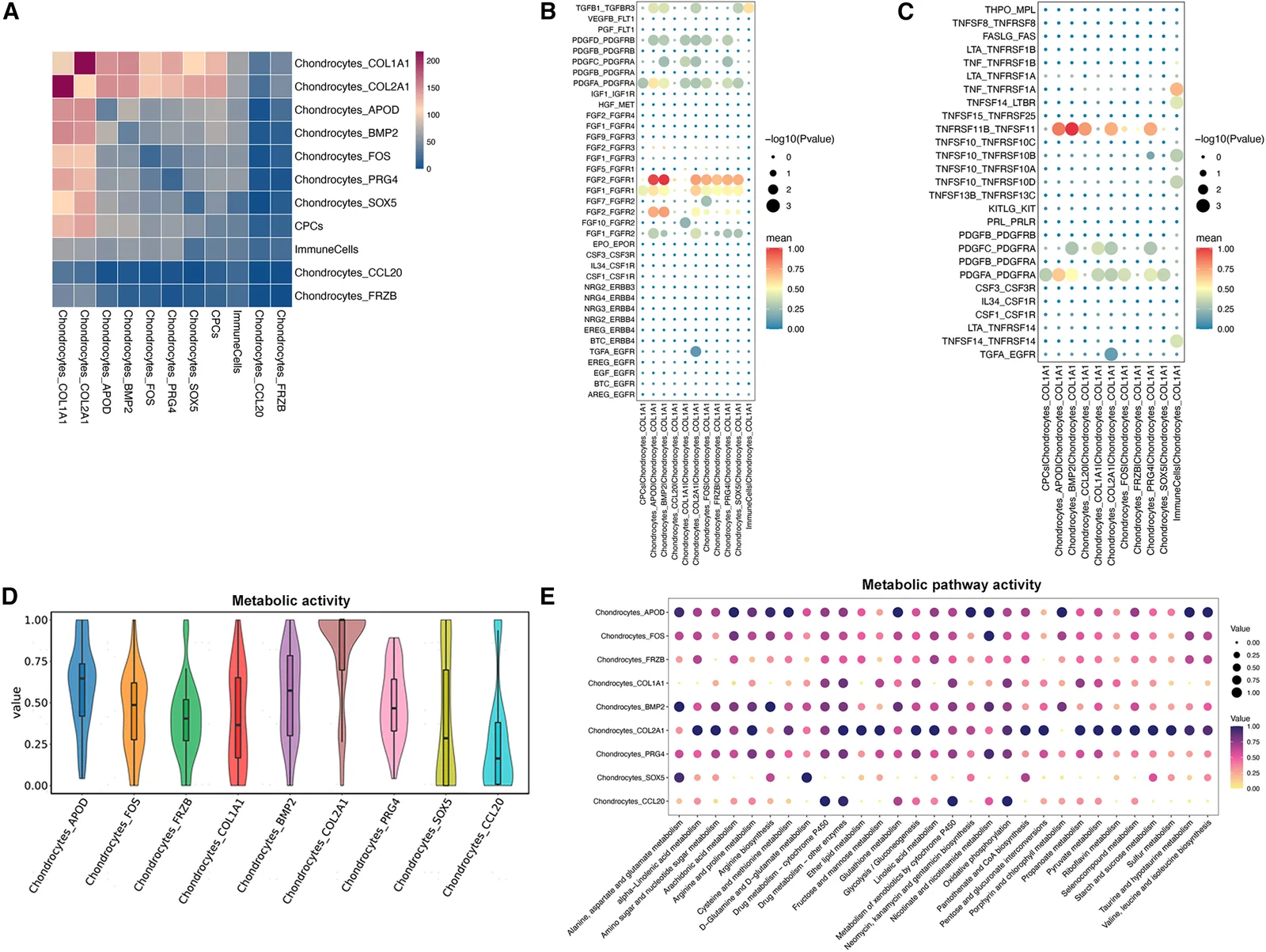
Intercellular communication and metabolic analysis of each chondrocyte subgroup were identified by scRNA-seq (A) Heatmap showing interaction logarithms between chondrocyte subgroups. (B) Bubble plots of predicted interactions between subgroups of chondrocytes as growth factor receptor cells. (C) Bubble plots of predicted interactions between subgroups of chondrocytes as cytokine receptor cells. (D) Metabolic activity of each chondrocyte subgroup. (E) Top 30 metabolic pathway activities in individual chondrocyte subgroups.

These findings highlight the distinct transcriptomic features of each chondrocyte subtype in ONFH. Furthermore, this study identified potential signaling pathways implicated in cellular communication between the chondrocyte subtypes. Heatmaps depicting interactions among all cell types highlighted significant ligand-receptor pairs for Chondrocytes_COL2A1 and Chondrocytes_COL1A1, which also interacted with other cell types, including Chondrocytes_APOD, Chondrocytes_BMP2, Chondrocytes_FOS, Chondrocytes_PRG4, Chondrocytes_SOX5, and CPCs (Figure 6A). The analysis of growth factor and cytokine interaction pairs focused on the interactions between Chondrocytes_COL1A1 and other cell types in ONFH (Figures 6B and 6C). Results revealed that fibroblast growth factor 2 (FGF2)-fibroblast growth factor receptor 1 (FGFR1) growth factor pairs were highly expressed between Chondrocytes_COL1A1 and Chondrocytes_APOD, as well as between Chondrocytes_COL1A1 and Chondrocytes_BMP2. Additionally, significant interactions involving the TNFRSF11B-TNFSF11 cytokine pair were identified between Chondrocytes_COL1A1 and multiple other cell types, suggesting its potential role in intercellular communication within cartilage tissue.

Lastly, analyses of metabolic activity and metabolic pathway activity (MPA) provided insights into the functional differences among the chondrocyte subtypes in ONFH. The results demonstrated that Chondrocytes_COL2A1 exhibited the highest metabolic activity, with increased participation in glycolysis/gluconeogenesis and oxidative phosphorylation pathways, indicating a higher rate of energy metabolism in this subpopulation (Figure 6D and 6E).

## Discussion

In addition to fat accumulation, osteoclast necrosis, and microvascular embolization, cartilage degeneration and damage play a pivotal role in the pathological changes associated with ONFH. Chondrocytes are also key target cells in the pathogenesis of ONFH. Apoptosis of chondrocytes, matrix degradation, and restricted repair contribute to femoral head collapse and joint deformation.^15^ Although scRNA-seq analyses of endothelial cells and adipocytes in ONFH have been reported, transcriptomic characterization of chondrocytes in the femoral head at single-cell resolution is lacking.^16^^,^^17^ This study is the first to identify the role of chondrocytes in ONFH using scRNA-seq, enabling a comprehensive identification of key markers and signaling pathways involved in the disease.

This study systematically delineates the cellular landscape of femoral head cartilage in osteochondritis dissecans (ONFD) using single-cell transcriptomics, revealing—for the first time—the pronounced heterogeneity of chondrocytes and the pivotal roles of two key disease-associated subpopulations, namely, the fibrotic (Chondrocytes_COL1A1) and reparative (Chondrocytes_COL2A1) subsets, in ONFD progression. Beyond chondrocytes, non-chondrocytic populations also display distinct molecular alterations. Specifically, CPCs overexpress the senescence marker Stmn1, whereas immune cells exhibit enrichment of Ptprc (CD45) and CD52 (Figure 2D). Previous studies have demonstrated that the STMN1-IGFBP5 axis promotes nucleus pulposus cell senescence and ECM degradation,^18^ while Ptprc and CD52 are implicated in immune cell infiltration and the regulation of T cell activation, respectively.^19^ Dysfunction of these non-chondrocytic populations may interact with pathologically altered chondrocytes through paracrine signaling, thereby synergistically exacerbating the degenerative microenvironment.

Pseudotime and PAGA analyses revealed that chondrocytes differentiate from the Chondrocytes_CCL20 precursor state along two distinct lineage trajectories: the CCL20→PRG4→COL1A1 fibrotic branch and the CCL20→SOX5→COL2A1 reparative branch (Figures 5A and 5B). As an early differentiation node, elevated CCL20 expression is closely associated with cartilage injury and inflammatory responses.^14^^,^^20^ SOX5, a well-established transcriptional regulator of chondrocyte differentiation and type II collagen synthesis, directs cells toward a reparative phenotype upon activation.^21^ PRG4, which is highly expressed in response to transforming growth factor β (TGF-β) stimulation, mechanical stress, and inflammatory factors, marks cells entering a pro-fibrotic intermediate state.^22^ This trajectory framework provides a fate-decision-level explanation for the coexisting pathological features of cartilage degeneration and repair in ONFH.

Immunohistochemistry further provided spatial evidence: COL1A1 was markedly upregulated throughout the deep cartilage layer, whereas COL2A1 overexpression was restricted to the superficial layer (Figure 4). These findings suggest irreversible fibrosis in the deep layer and a transitional “repair-inflammation” state in the superficial layer.^6^ Inter-sample variation in the COL1A1/COL2A1 ratio (Figure 3B) suggests that this ratio dynamically reflects the progression of necrosis: a high ratio indicates dominance of advanced fibrosis, whereas relatively elevated COL2A1 expression points to a disease stage in which reparative processes remain active.

To functionally validate these findings, we established an *in vitro* ONFH chondrocyte injury model using DEX treatment. Immunofluorescence analysis confirmed that DEX treatment significantly upregulated COL1A1 and COL2A1 protein expression (Figures 4I–4M), faithfully recapitulating the aberrant expression patterns observed in clinical specimens and transcriptomic data. Quantitative real-time PCR revealed that transcription factors characteristic of the COL1A1^+^ subpopulation (e.g., EBF1, RUNX2, and STAT2) and those of the COL2A1^+^ subpopulation (e.g., FOXA3, RARG, and HOXA2) were concomitantly upregulated upon pathological stimulation (Figures 4N and 4O), a finding highly consistent with the regulator predictions from single-cell analysis. Notably, RUNX2—a key driver of chondrocyte hypertrophy and MMP13 transcription^23^^,^^24^—exhibited functional coupling with the focal adhesion and ECM-receptor interaction pathways enriched in the COL1A1^+^ subpopulation (Figure 3C).^25^ This heightened activity suggests that RUNX2 may serve as a pivotal node linking ECM mechanical sensing, Hippo-YAP pathway activation, and matrix degradation.^26^

Intercellular communication analysis revealed that Chondrocytes_COL1A1 functions as a central hub for fibroblast growth factor (FGF) and tumor necrosis factor (TNF) superfamily signaling. Specifically, it receives FGF2-FGFR1 signals from Chondrocytes_APOD and Chondrocytes_BMP2, while also exhibiting high expression of the TNFRSF11B-TNFSF11 (OPG-RANKL) ligand-receptor pair (Figures 6B and 6C). Chronic overexpression of FGF2 induces chondrocyte hypertrophy and catabolic metabolism,^27^ whereas RANKL signaling promotes MMP expression and ECM degradation through the nuclear factor (NF)-κB pathway.^28^ These signaling events act in synergy with RUNX2 activity, conferring a sustained catabolic phenotype upon this subpopulation. Metabolic analysis further revealed that Chondrocytes_COL2A1 exhibits the highest overall metabolic activity, with significant enrichment in glycolysis and oxidative phosphorylation pathways (Figures 6D and 6E). This metabolic profile supplies the energy and substrates required for type II collagen synthesis and proteoglycan assembly,^6^ accounting for its niche role as either a source of specific ligands or a signaling responder. Cross-referencing the cell-autonomous enrichment profiles (Figure 3) with the signaling network (Figure 6) provided bidirectional validation: the COL1A1^+^ subpopulation exhibited KEGG pathway enrichment in “antigen processing and presentation” and “growth factor binding,” while also serving as a key node in TNF and FGF signaling. This finding indicates that the COL1A1^+^ subpopulation not only harbors an intrinsic pro-inflammatory transcriptional program but also actively contributes to the construction and amplification of the local inflammatory microenvironment.

By integrating multidimensional analyses, this study proposes a regulatory network model for ONFH chondrocytes that encompasses fate determination, transcriptional regulation, metabolism, intercellular communication, and functional output. In this model, Chondrocytes_CCL20 serves as the differentiation starting point, with PRG4 and SOX5 directing cells toward fibrotic and reparative lineages, respectively. The COL1A1^+^ subpopulation, driven by RUNX2 and Hippo-YAP pathways, exhibits hallmark pathway activities such as ECM degradation, focal adhesion, and antigen processing, and interacts with the microenvironment through the FGF2-FGFR1 and TNFRSF11B-TNFSF11 axes. The COL2A1^+^ subpopulation is characterized by the high activity of transcription factors, including FOXA3 and RARG, which support its synthetic and reparative functions through elevated metabolic energy production. This model was preliminarily validated in the *in vitro* DEX-induced model at both the protein expression and transcriptional regulation levels, thereby providing an integrated multi-omics evidence chain for the “fibrosis-repair” dual imbalance underlying ONFH cartilage degeneration.

### Limitations of the study

This study has several limitations. First, the sample size for single-cell sequencing was relatively modest (10 ONFH patients and 10 controls), and there was an age discrepancy between the two groups, which is inherent to the distinct epidemiological profiles of the two conditions. Second, an ONFH animal model for *in vivo* functional validation was not established, and the mechanistic analysis of the Hippo-YAP signaling pathway and its associated transcription factors remain incomplete. Third, the proposed regulatory network is primarily derived from bioinformatics analyses and *in vitro* experiments; thus, its causal relationships require confirmation through subsequent functional studies. Finally, all ONFH patients were at Ficat stage III (collapse stage), and the study did not capture the cellular and molecular changes occurring during the early stages of the disease. In this study, we systematically charted the cellular heterogeneity of ONFH cartilage using single-cell transcriptomics and identified nine distinct chondrocyte subpopulations. Among these, Chondrocytes_COL1A1 and Chondrocytes_COL2A1 emerged as key subpopulations specifically enriched in ONFH. The COL1A1^+^ subpopulation is characterized by fibrotic, pro-inflammatory, and matrix-degrading features, with activation of the Hippo-YAP pathway driven by the core transcription factor RUNX2. In contrast, the COL2A1^+^ subpopulation exhibits high metabolic activity and a synthetic, reparative phenotype regulated by transcription factors such as FOXA3 and RARG. Pseudotime analysis revealed that CCL20^+^ progenitor cells differentiate along fibrotic or reparative trajectories via PRG4 or SOX5, respectively. Cell-communication analysis further elucidated the pivotal roles of the FGF2-FGFR1 and TNFRSF11B-TNFSF11 axes in mediating interactions between the COL1A1^+^ subpopulation and its microenvironment. *In vitro* functional validation confirmed the robust reproducibility of these molecular signatures in a DEX-induced chondrocyte injury model. Collectively, our findings uncover the cellular and molecular mechanisms underlying the “fibrosis-repair” dual imbalance in ONFH cartilage degeneration, thereby offering potential therapeutic targets and a theoretical framework for modulating chondrocyte fate and intervening in the pathological microenvironment.

## Resource availability

### Lead contact

Further information and requests for resources and reagents should be directed to and will be fulfilled by the lead contact, Jidong Song (sjidong12@xjtu.edu.cn).

### Materials availability

This study did not generate new unique reagents.

### Data and code availability

• Single-cell RNA sequencing data have been deposited at GEO: GSE311084 and are publicly available as of the date of publication.
• This paper does not report original code.
• Any additional information required to reanalyze the data reported in this paper is available from the lead contact upon request.

## Acknowledgments

We are grateful to all authors for their dedicated participation in the current study. The present study was supported by the 10.13039/501100001809National Natural Science Foundation of China (grant no. 82102566).

## Author contributions

T.L., formal analysis, investigation, validation, writing – original draft; K.N., formal analysis, methodology, resources, writing – review and editing; X.D., writing – review and editing; W.W., conceptualization, investigation, methodology, supervision; J.S., conceptualization, investigation, methodology, resources, supervision, writing – original draft, writing – review and editing.

## Declaration of interests

All authors declare that they have no conflict of interest.

## Declaration of generative AI and AI-assisted technologies in the writing process

During the preparation of this work, the authors used ChatGPT in order to improve the language and readability of the manuscript. After using this tool/service, the authors reviewed and edited the content as needed and take full responsibility for the content of the published article. No AI tools were used for figure generation, image processing, or data analysis.

## STAR★Methods

### Key resources table

**Table:** 

REAGENT or RESOURCE	SOURCE	IDENTIFIER
**Antibodies**		

COL1A1	Servicebio	GB115707-100
COL2A1	Servicebio	GB115732-100

**Biological samples**		

Human hip joint cartilage	The Second Affiliated Hospital, Xi’an Jiaotong University	This paper

**Deposited data**		

Single-cell RNA sequencing data	This paper	GEO: GSE311084

**Experimental models: Cell lines**		

Primary rat chondrocytes	SD rats	N/A

**Oligonucleotides**		

BHLHE22(Forwardprimer: CTACCCCTTTAGTGCAGGGC, Reverseprimer: CTTCTCCGTGCACTGTTTGC)	This paper	N/A
EBF1 (Forwardprimer: GGGATCCAGGAAAGCATCCA, Reverseprimer: GCTGCTTCTCAAAGTGAGCC)	This paper	N/A
MEIS3 (Forwardprimer: GGGGATCTTCCCCAAAGTGG, Reverseprimer: ATCATGGGCTGCACTATCCG)	This paper	N/A
RUNX2 (Forwardprimer: AGTCAGATTACAGATCCCAGGC, Reverseprimer: GAGGTGGCAGTGTCATCATCTG)	This paper	N/A
STAT2 (Forwardprimer: CGCATCATGGGCTTTGTGAG, Reverseprimer: ACCCAAGAGCAGGTAATGCC)	This paper	N/A
COL1A1 (Forwardprimer: TGGCAAGAACGGAGATGA, Reverseprimer: AGCTGTTCCAGGCAATCC)	This paper	N/A
COL2A1 (Forwardprimer: GCAACAGCAAGGTCTTGCTG, Reverseprimer: GAGGTCTTGCGGAGGTTCTG)	This paper	N/A
FOXA3 (Forwardprimer: GAGTGGAGCTACTACCCGGA, Reverseprimer: GGTAGGGAGAGCTAAGAGGGT)	This paper	N/A
RARG (Forwardprimer: CGGGTCCTTGGTGTTCTAGC, Reverseprimer: TTGTAGACTGGCAGGCTGTG)	This paper	N/A
HOXA2 (Forwardprimer: AAGCCCCTCCAAAAGAGGGA, Reverseprimer: AGTGTCGAGTGTGAAAGCGT)	This paper	N/A
POLR3G (Forwardprimer: CTGCAGTGAGCGAAGAGCTA, Reverseprimer: CGAGGAACAGGCGATCTGG)	This paper	N/A
FOXB1 (Forwardprimer: GTACAAGATGCCTGGGGGAC, Reverseprimer: CTCCATAGGCGCTGTAGTCG)	This paper	N/A

**Software and algorithms**		

Featurecounts v2.0.1	subread	http://subread.sourceforge.net
CeleScope v1.5.2	Singleron	https://singleron.bio/products/celescope/
STAR(v2.6.1b)	N/A	https://alexdobin.github.io/STAR/
Scanpy v1.8.1	N/A	https://scanpy.scverse.org/en/stable/

### Experimental model and study participant details

A total of 20 subjects undergoing total hip arthroplasty (THA) provided hip cartilage samples for this study. The ONFH group consisted of 10 patients with Ficat stage III idiopathic femoral head necrosis (age range: 47–57 years), while the normal control group comprised 10 sex-matched patients with acute femoral neck fractures (age range: 59–65 years) who underwent THA within 24 h of injury. All cartilage samples were harvested from the anterosuperior region of the femoral head within 2 h post-operatively (from the collapsed site in the ONFH group) and screened for macroscopic integrity and graded according to the Histologic-Histochemical Grading System (HHGS); specimens with HHGS > grade 2 or grossly damaged appearance were excluded. Ultimately, 6 samples were used for single-cell sequencing (3 from the ONFH group and 3 from the control group), while another 10 were used for immunohistochemical validation (5 from the ONFH group and 5 from the control group); a total of 4 samples were excluded after quality control. The two groups were matched 1:1 by sex; however, due to the limited sample size, no subgroup analysis by sex was performed, and the data did not indicate a significant effect of sex on chondrocyte subset distribution or key molecular expression patterns. To validate the single-cell sequencing findings *in vitro*, primary chondrocytes were isolated from the articular cartilage of the femoral condyles and tibial plateaus of 7- to 10-day-old Sprague-Dawley rats (both sexes) and treated with dexamethasone (10^−4^ M for 48 h) to establish an *in vitro* chondrocyte injury model. All human sample protocols were approved by the Ethics Committee of The Second Affiliated Hospital of Xi’an Jiaotong University (approval no. 2017-196), and written informed consent was obtained from all participants. All animal experiments were approved by the Animal Ethics Committee of Xi’an Jiaotong University (approval no. 2021-1261). Detailed subject information is provided in Table S1.

### Method details

#### Tissue dissociation and single-cell preparation

The experimental workflow is depicted in Figure 1 and Figure S2. The cartilage tissues were preserved in the GEXSCOPE Tissue Preservation Solution (Singleron) and transported to the Singleron lab on ice. The specimens were washed 3 times with Hanks Balanced Salt Solution and shred into 1–2 mm pieces. The tissue debris was then digested with 2 mL GEXSCOPE Tissue Dissociation Solution (Singleron) at 37°C for 15 min with agitation. Cells were filtered through 40-micron sterile strainers (Falcon) and centrifuged at 300 g for 5 min. After discarding the supernatant, the pellets were resuspended in 1 mL PBS. Red blood cells were removed by adding 2 mL RBC lysis buffer (Roche) followed by centrifugation at 500 × g for 5 min and resuspend in PBS. Finally, a sample of the cell mixture was stained with trypan blue (Bio-RAD) for cell viability assessment under the microscope.

#### ScRNA-seq library construction and preliminary results

Single-cell suspensions containing 1×10^5^ cells/mL in PBS were prepared and then loaded onto microfluidic devices for scRNA-seq library construction, following the Singleron GEXSCOPE protocol. Individual libraries were diluted to 4 nM and pooled for sequencing on an Illumina HiSeq X with 150 bp paired end reads. Raw reads underwent quality control processing using fastQC and fastp to eliminate low-quality reads. Poly-A tails and adaptor sequences were removed by cutadapt. Subsequentely, the processed reads were mapped to the reference genome GRCh38 (ensembl version 92 annotation) using STAR. Gene counts and unique molecular identifier (UMI) counts were obtained by featureCounts software. Expression matrix files for further analyses were created based on gene counts and UMI counts.

#### Single-cell sequencing data analysis

Raw reads were processed to generate gene expression profiles using CeleScope v1.5.2 (Singleron) with default parameters. Briefly, Barcodes and UMIs were extracted from R1 reads and corrected. Adapter sequences and poly A tails were trimmed from R2 reads and the trimmed R2 reads were aligned against the GRCh38 (hg38) transcriptome using STAR (v2.6.1 b). Uniquely mapped reads were then assigned to exons with FeatureCounts (v2.0.1). Successfully Assigned Reads with the same cell barcode, UMI and gene were grouped together to generate the gene expression matrix for further analysis.

#### Quality control, dimension-reduction and clustering

Scanpy v1.8.1 was used for quality control, dimensionality reduction and clustering under Python 3.7. For each sample dataset, we filtered expression matrix by the following criteria: 1) cells with gene count less than 200 or with top 2% gene count were excluded; 2) cells with top 2% UMI count were excluded; 3) cells with mitochondrial content >20% were excluded; 4) genes expressed in less than 5 cells were excluded. The raw count matrix was normalized by total counts per cell and logarithmically transformed into normalized data matrix. Top 2000 variable genes were selected by setting flavor = ‘seurat’. Principal Component Analysis (PCA) was performed on the scaled variable gene matrix, and top 20 principle components were used for clustering and dimensional reduction. Cells were separated into 18 clusters by using Louvain algorithm and setting resolution parameter at 1.2. Cell clusters were visualized by using Uniform Manifold Approximation and Projection (UMAP).^29^

#### Differentially expressed genes (DEGs) analysis

DEG analysis was performed with the scanpy.tl.rank_genes_groups function based on Wilcoxon rank-sum test with default parameters, and genes expressed in more than 10% of the cells in either of the compared groups of cells and with an average log(Fold Change) value greater than 1 were selected as DEGs. Adjusted *p* value was calculated by benjamini-hochberg correction and the value 0.05 was used as the criterion to evaluate the statistical significance.

#### Go and KEGG enrichment

Gene Ontology (GO) and Kyoto Encyclopedia of Genes and Genomes (KEGG) analysis were used with the “clusterProfiler” R package v 3.16.1.^30^ Pathways with p_adj value less than 0.05 were considered as significantly enriched. Selected significant pathways were plotted as bar plots.

#### Celltype identification

Hypergeometric tests (HGT) were performed on these gene sets against cartilage reference from SynEcoSys database, which contains all cell-type’s featured genes in the specific organ/tissue.^31^^,^^32^ Identity of each cell was determined as the cell-type has the minimal HGT *p* value. For cluster annotation, Frequency of each cell-type was calculated in each cluster, and cell-type with highest frequency was chosen as cluster’s identity.The cell type identification of each cluster was determined according to the expression of canonical markers from the reference database SynEcoSysTM (Singleron Biotechnology). SynEcoSysTM contains collections of canonical cell type markers for single-cell seq data, from CellMakerDB, PanglaoDB and recently published literatures. To obtain a high-resolution map of chondrocytes, cells from the specific cluster were extracted and reclustered for more detailed analysis following the same procedures described above and by setting the clustering resolution as 0.5.

#### Filtering cell doublets

Cell doublets were estimated based on the expression pattern of canonical cell markers. Any clusters enriched with multiple cell type-specific markers were excluded for downstream analysis.

#### Pseudotime trajectory analysis

Cell differentiation trajectory of monocyte subtypes was reconstructed with the Monocle2 v 2.10.0. For constructing the trajectory, top 2000 highly variable genes were selected by Seurat (v3.1.2) FindVairableFeatures function, and dimension-reduction was performed by DDRTree function. The trajectory was visualized by plot_cell_trajectory function in Monocle2.^33^

#### Immunohistochemical assays

Human femoral head specimens were fixed in 4% paraformaldehyde for 72 h and decalcified in EDTA solution (pH 7.4) for 40 days. The tissues were then dehydrated through a graded ethanol series (75%–100%), cleared in xylene, and embedded in paraffin. Sections of 3–5 μm thickness were prepared, baked at 60°C for 4 h, dewaxed in xylene, and rehydrated through a descending ethanol series. Antigen retrieval was carried out using pepsin solution at 37°C for 10–30 min. Endogenous peroxidase activity was quenched with 3% H_2_O_2_, and nonspecific binding sites were blocked with 10% normal goat serum. The sections were incubated overnight at 4°C with primary antibodies against COL1A1 (1:500, GB115707-100, Servicebio) and COL2A1 (1:500, GB115732-100, Servicebio), followed by incubation with an HRP-conjugated secondary antibody (1:100) at room temperature for 60 min. Detection was performed using DAB substrate, and nuclei were counterstained with hematoxylin. Finally, the sections were dehydrated, cleared, and mounted with neutral resin.Immunohistochemical staining was evaluated semi-quantitatively by two independent investigators in a blinded manner. Staining was scored based on the percentage of positive cells as follows: 0 (<5%), 1 (5%–25%), 2 (26%–50%), 3 (51%–75%), or 4 (>75%).

#### Immunofluorescence staining

Primary rat chondrocytes seeded on glass coverslips were treated with vehicle or 10^−4^ M dexamethasone for 48 h. Cells were fixed with 4% paraformaldehyde for 15 min, permeabilized with 0.1% Triton X-100 for 10 min, and blocked with 5% bovine serum albumin (BSA) for 1 h at room temperature. The coverslips were then incubated overnight at 4°C with primary antibodies against COL1A1 (Abmart, P28372, 1:50) and COL2A1 (Affinity, AF0135, 1:50). After washing, cells were incubated with FITC-conjugated secondary antibody (Jackson ImmunoResearch, 1:200) for 1 h at room temperature in the dark. Nuclei were counterstained with DAPI. Fluorescence images were captured using a laser confocal microscope (Zeiss, Germany), and mean fluorescence intensity was quantified using ImageJ software.

#### Quantitative real-time PCR (q-PCR)

Total RNA was isolated from cultured chondrocytes using TRIzol reagent (AG Scientific, China) and reverse-transcribed into cDNA using the PrimeScript RT reagent Kit (Takara, Japan) according to the manufacturer’s instructions. qRT-PCR was performed on a QuantStudio 5 system (Thermo Fisher, USA) using TB Green Premix Ex Taq (Takara, Japan). Relative gene expression was calculated using the 2^−^ΔΔCt method with β-actin as the internal reference.

### Quantification and statistical analysis

Differential expression analysis of single-cell RNA sequencing data was performed using the Wilcoxon rank-sum test. *p*-values were adjusted using the Benjamini-Hochberg method, with statistical significance defined as adjusted *p* < 0.05 and |log_2_FC| > 1. GO and KEGG enrichment analyses were performed using the clusterProfiler R package (v3.16.1), with adjusted *p* < 0.05 considered significant. Intergroup comparisons for immunohistochemistry were performed using the Mann-Whitney U test, while immunofluorescence and qRT-PCR data were analyzed by unpaired Student’s *t* test. All *in vitro* experiments were independently repeated three times, and data are expressed as mean ± standard error of the mean (SEM). Statistical significance was set at *p* < 0.05. Detailed statistical information, including sample size (n), what n represents, exact *p*-values, and precision measures, is provided in the corresponding figure legends and Results section.
