# Supplementary material for: Single-cell RNA sequencing reveals chondrocyte heterogeneity and fibrocartilage transition in osteonecrosis of the femoral head
Source: iScience. 2026 Aug 22;29(9):116724. doi: 10.1016/j.isci.2026.116724 (PMC13524726; doi:10.1016/j.isci.2026.116724)
Supplement: Supplementary file 1 [file mmc1.pdf]

iScience, Volume *29*

Supplemental information

Single-cell RNA sequencing reveals chondrocyte heterogeneity and fibrocartilage transition in osteonecrosis of the femoral head

Tun Liu, Kai Nan, Xiaoqian Dang, Wei Wang, and Jidong Song

Supplementary information

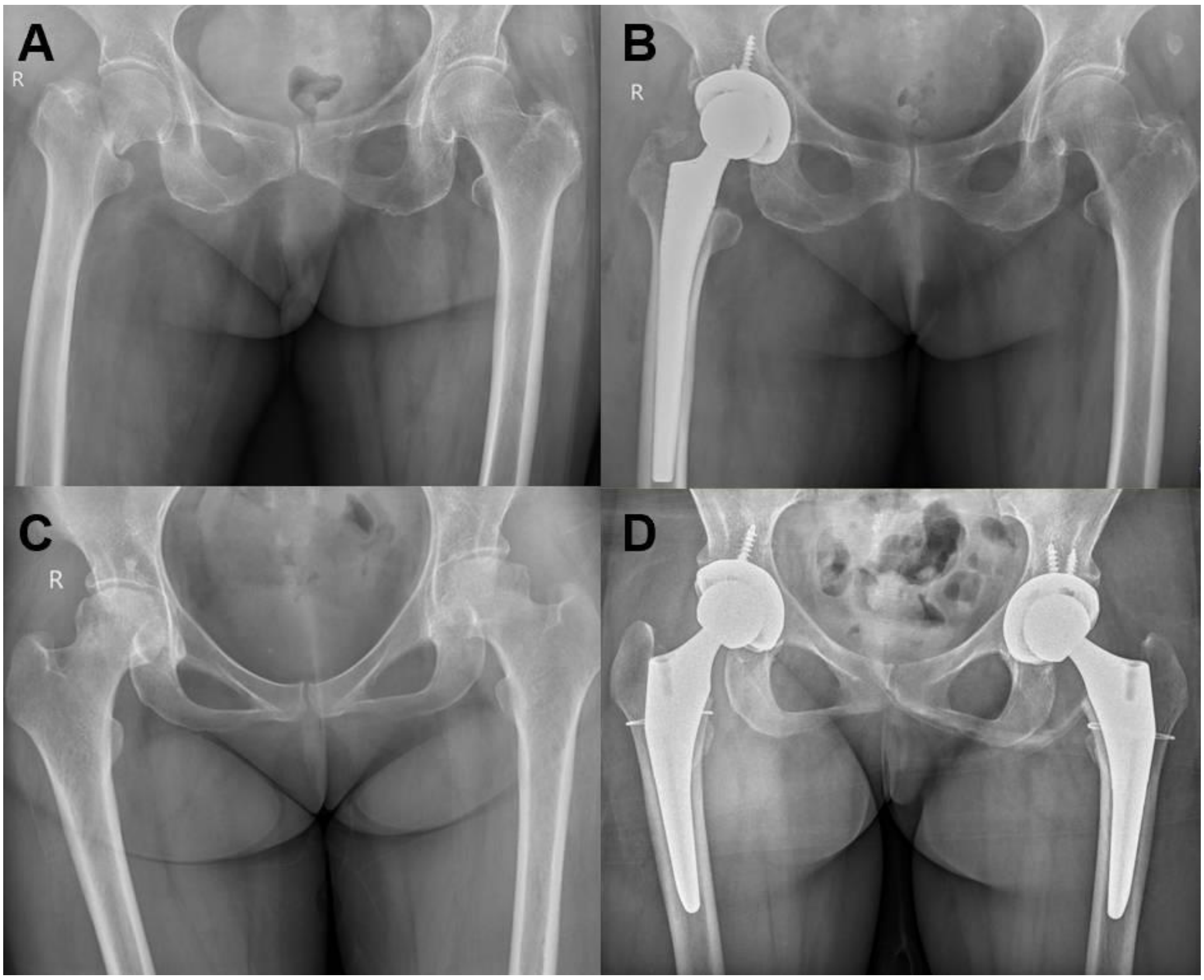

Fig S1. Typical X-ray images of patients in this study. **A and B** Preoperative and postoperative X-rays of patients with femoral neck fractures. **C and D** Preoperative and postoperative X-rays of patients with ONFH.

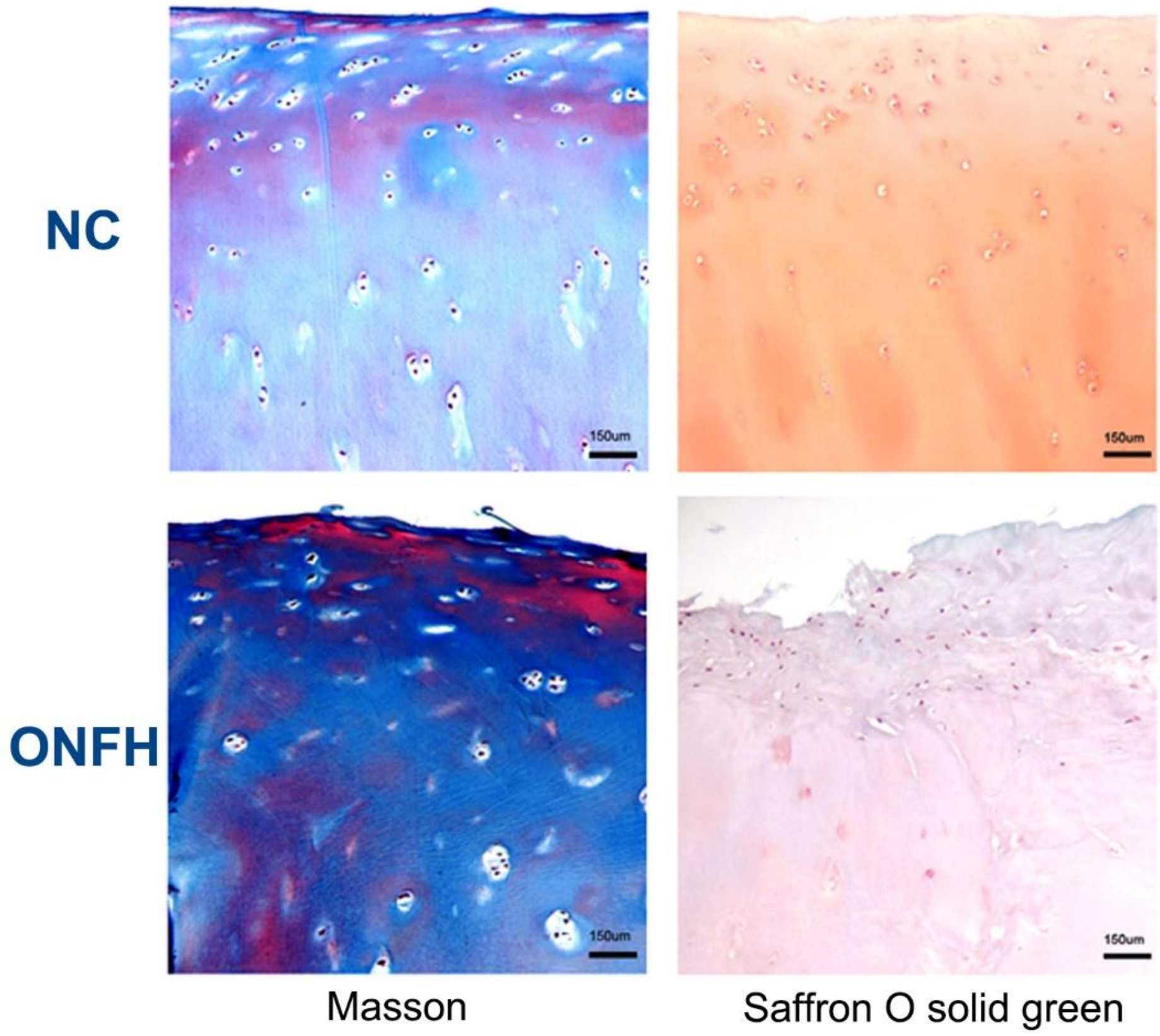

Fig S2. Characteristic cartilage staining of femoral head specimens. Masson and Saffron O solid green stained sections of femoral head cartilage from normal controls and patients with primary hip osteonecrosis. Scale bar, 150 μm.

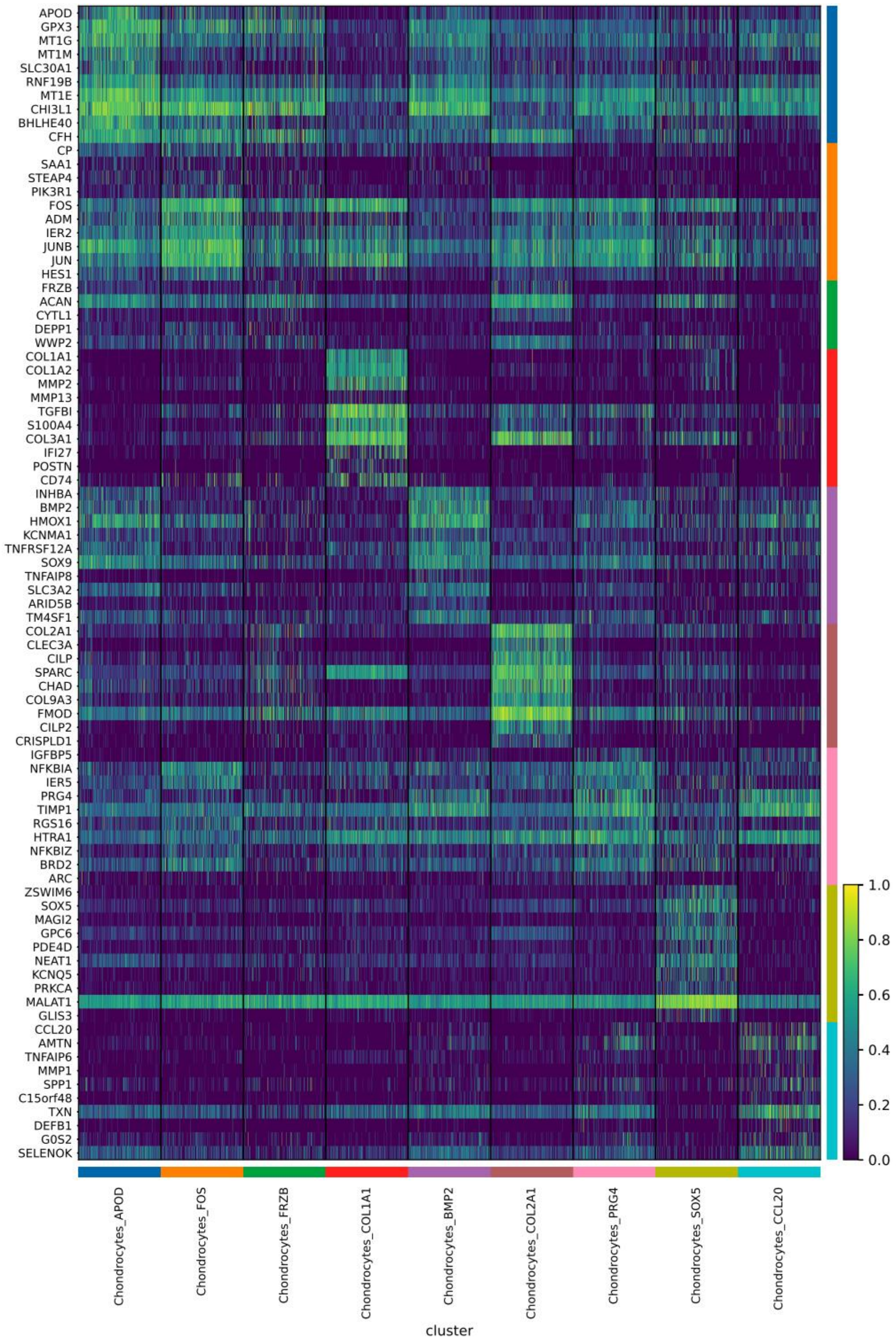

Fig S3. The heatmap for the annotations of the 9 most critical subpopulations.

TableS1 Basic characteristics of the subjects in this study

Group	NC		ONFH		
	Age (years)	Gender	Age (years)	Gender	Ficat stage
Single-cell sequencing					
**1**	60	Male	52	Male	III
**2**	61	Male	50	Female	III
**3**	59	Female	56	Male	III
Immunohistochemical assays					
**4**	62	Male	47	Male	III
**5**	62	Male	52	Male	III
**6**	63	Male	54	Male	III
**7**	65	Female	54	Female	III
**8**	60	Female	53	Female	III
**9**	61	Female	57	Female	III
**10**	65	Female	57	Female	III

TableS2 Cell numbers and proportion in each group

Cluster	NC	ONFH
**CPCs**	0(0.00%)	382(1.68%)
**Chondrocytes**	28087(100.00%)	22301(97.97%)
**ImmuneCells**	0(0.00%)	81(0.36%)
**SUM**	28087	22764

TableS3 Cell numbers and proportion in each sample

Cluster	NC_1	NC_2	NC_3	ONFH_1	ONFH_2	ONFH_3
**CPCs**	0(0.00%)	0(0.00%)	0(0.00%)	99(1.22%)	36(0.41%)	247(4.23%)
**Chondrocytes**	5558(100.00%)	13615(100.00%)	8914(100.00%)	8033(98.76%)	8679(98.70%)	5589(95.75%)
**ImmuneCells**	0(0.00%)	0(0.00%)	0(0.00%)	2(0.02%)	78(0.89%)	1(0.02%)
**SUM**	5558	13615	8914	8134	8793	5837
